# A retrospective analysis exploring the association of pretreatment neutrophil-to-lymphocyte ratio and immune checkpoint inhibitor outcomes in patients with advanced NSCLC and liver metastases

**DOI:** 10.1177/17588359251367315

**Published:** 2025-10-22

**Authors:** Maisam Makarem, Tuan Hoang, Mitchell J. Elliott, Andrew Rabinovitch, Katrina Hueniken, Shelley Kuang, Sabine Schmid, Ming Sound Tsao, Tracy McGaha, Pamela S. Ohashi, Frances A. Shepherd, Penelope A. Bradbury, Geoffrey Liu, Natasha B. Leighl, Adrian Sacher, Sally C. M. Lau

**Affiliations:** Princess Margaret Cancer Centre, University Health Network, Toronto, ON, Canada; Princess Margaret Cancer Centre, University Health Network, Toronto, ON, Canada; Princess Margaret Cancer Centre, University Health Network, Toronto, ON, Canada; Princess Margaret Cancer Centre, University Health Network, Toronto, ON, Canada; Princess Margaret Cancer Centre, University Health Network, Toronto, ON, Canada; Department of Biostatistics, University Health Network, Toronto, ON, Canada; Princess Margaret Cancer Centre, University Health Network, Toronto, ON, Canada; Princess Margaret Cancer Centre, University Health Network, Toronto, ON, Canada; Department of Medical Oncology, Inselspital, University Hospital Bern, Bern, Switzerland; Princess Margaret Cancer Centre, University Health Network, Toronto, ON, Canada; Department of Laboratory Medicine and Pathobiology, University of Toronto, Toronto, ON, Canada; Princess Margaret Cancer Centre, University Health Network, Toronto, ON, Canada; Department of Immunology, University of Toronto, Toronto, ON, Canada; Princess Margaret Cancer Centre, University Health Network, Toronto, ON, Canada; Department of Immunology, University of Toronto, Toronto, ON, Canada; Princess Margaret Cancer Centre, University Health Network, Toronto, ON, Canada; Princess Margaret Cancer Centre, University Health Network, Toronto, ON, Canada; Princess Margaret Cancer Centre, University Health Network, Toronto, ON, Canada; Princess Margaret Cancer Centre, University Health Network, Toronto, ON, Canada; Princess Margaret Cancer Centre, University Health Network, 610 University Ave., Toronto, ON M5G 2M9, Canada; Department of Immunology, University of Toronto, Toronto, ON, Canada; Princess Margaret Cancer Centre, University Health Network, 610 University Ave., Toronto, ON M5G 2M9, Canada

**Keywords:** immune checkpoint inhibitors, liver metastases, NLR, NSCLC

## Abstract

**Background:**

Liver metastases (LM) in advanced non-small-cell lung cancer (NSCLC) are associated with poor clinical outcomes. The tolerogenic immune microenvironment in the liver may contribute to inferior response to immune checkpoint inhibitors (ICIs). We hypothesized that the presence of LM may be associated with an expanded peripheral myeloid population, using the neutrophil-to-lymphocyte ratio (NLR) as a surrogate in patients treated with ICIs.

**Objectives:**

We evaluated the impact of LM and NLR on clinical outcomes in patients with advanced NSCLC treated with ICIs.

**Design:**

This was a retrospective analysis conducted at a single cancer center.

**Methods:**

We reviewed the records of 324 patients with advanced NSCLC treated with programmed death ligand-1 (PD-L1) inhibitors as monotherapy or in combination with cytotoxic T-lymphocyte antigen-4 (CTLA-4) inhibitors. Clinical outcomes, including progression-free survival (PFS) and overall survival (OS), were evaluated among patients with and without LM in NLR-High (NLR ⩾ 5) and NLR-Low (NLR < 5) subgroups.

**Results:**

Patients with LM had significantly higher median NLR compared to patients without LM (median 7.3 vs 4.5, *p* < 0.001). Patients with LM had significantly shorter median PFS (HR 1.54, *p* = 0.006) and median OS (HR 1.56, *p* = 0.014). Patients with LM who were NLR-High (LM+ NLR-H) had shorter median PFS and median OS (2.0 months and 5.4 months, respectively) compared to patients who had LM and were NLR-Low (LM+ NLR-L, median PFS 4.0 months and median OS 13.3 months). This trend was also observed within the PD-L1 > 50% subgroup. In 42 patients with evaluable response and LM, 25/42 (59.5%) of patients had concordant responses in the liver and extra-hepatic sites.

**Conclusion:**

Patients with LM who are NLR-High had the poorest clinical outcomes to ICI, and this appeared to be irrespective of PD-L1 status. Ongoing translational work will provide further insight into tumor myeloid subpopulations that may correlate with treatment resistance.

## Plain language summary

Liver metastases and blood inflammatory marker NLR predict patient outcomes with immunotherapy in lung cancer treatment

In patients with advanced non-small cell lung cancer (NSCLC), those with disease spread to the liver tend to respond poorly to immunotherapy. This study looked at the whether blood test values of neutrophil-to-lymphocyte ratios (NLR), reflecting inflammatory status, can help explain this. We reviewed data from 324 patients treated with immunotherapy and found that patients with liver metastases had higher NLRs and worse outcomes. In particular, patients with both liver metastases and high NLR had the shortest survival. These findings suggest that inflammation status, as reflected by NLR, may play a role in predicting poor response to immunotherapy in this group. Future studies will focus on further understanding at the molecular level.

## Graphical abstract

**Graphical Abstract:**
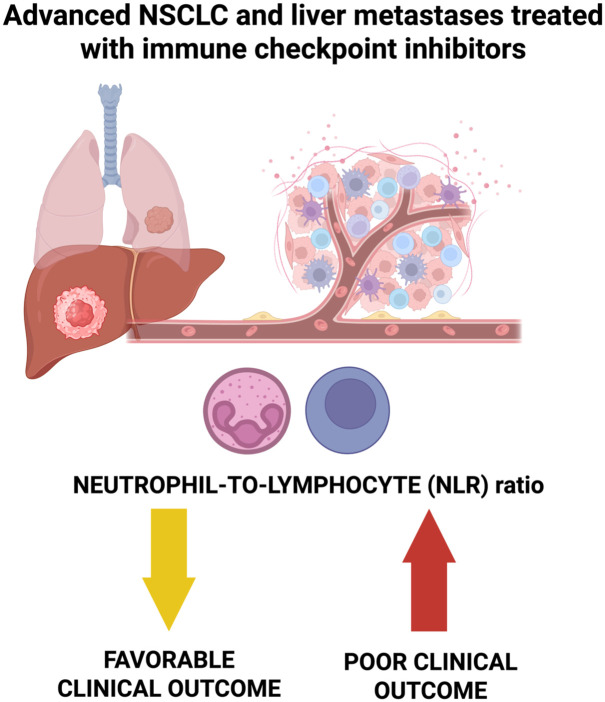

## Introduction

Over the past decade, immune-modulating therapies have become an integral treatment modality for patients with lung cancer. Immune checkpoint inhibitors (ICIs) alone or in combination with chemotherapy have demonstrated clinical efficacy in patients with early-stage and advanced non-small-cell lung cancer (NSCLC), where durable responses are seen.^1–5^ However, there is an ongoing need to characterize and understand patients who might derive greater or less benefit from ICI.

Several studies have demonstrated that patients with liver metastases (LM) have poor prognosis and have worse outcomes to systemic therapies, including ICI.^6–9^ Among patients with NSCLC treated with pembrolizumab monotherapy as part of KEYNOTE-001, a significantly lower objective response rate (ORR) and shorter median progression-free survival (PFS) were observed in patients with liver metastasis compared to those without (median PFS (mPFS) 1.8 months vs 4.0 months, *p* = 0.009).^8^ A pooled subgroup analysis of patients with NSCLC treated with durvalumab monotherapy on the ATLANTIC study and Study 1108 showed similar findings, with lower ORR among those with LM and worse OS(insert ref 9). The inherently tolerogenic nature of the liver microenvironment may explain the reduced efficacy of PD-(L)1 inhibitors in NSCLC patients with LM.^8,9^

Identifying patients who derive limited benefit from ICI is crucial for optimizing therapeutic strategies. Despite the challenges in establishing reliable predictive biomarkers for ICI response to date, exploring alternative markers can improve our ability to proactively plan treatment options. While programmed death ligand-1 (PD-L1) expression has served as a standard biomarker for predicting responses to anti-PD-1 or anti-PD-L1 therapies in NSCLC, ongoing research is delving into emerging predictive biomarkers. These include factors such as tumor mutation or neoantigen burden, T-cell receptor clonality, tumor infiltrating lymphocytes, and the neutrophil-to-lymphocyte ratio (NLR), among others.^10–12^ While tumor-infiltrating lymphocytes are vital for tumor defense and correlate with better prognosis, neutrophil recruitment by tumors is thought to promote angiogenesis and early tumor growth development and metastasis.^13^ NLR may also offer insight into the impact of the tumor on expansion of immunosuppressive myeloid populations both in the periphery as well as the tumor immune microenvironment.^14,15^ Recent studies have demonstrated that a low NLR predicts more favorable outcomes with ICI in patients with advanced NSCLC.^16–18^ In addition, the Lung Immune Prognostic Index, combining derived NLR and LDH, was shown to predict outcomes for patients with NSCLC treated with ICIs.^19,20^ Therefore, we sought to explore the potential of NLR as a biomarker for ICI response in patients with advanced NSCLC and LM.

## Methods

### Study population

A single-center retrospective analysis of patients with advanced NSCLC who received a PD-(L)1 inhibitor (PD-(L)1i) as monotherapy or in combination with a CTLA-4 inhibitor (CTLA-4i) between 2017 and 2022 at the Princess Margaret Cancer Centre, Toronto, Canada was conducted. Patients were excluded from the study if they had *EGFR*, *ALK*, *RET*, or *ROS1* alterations or received chemotherapy in combination with immunotherapy. Patients with adenocarcinoma histology completed next-generation sequencing, polymerase chain reaction (PCR), or immunohistochemistry-based genomic alteration assessment. Baseline absolute NLR was calculated using the complete blood count prior to initiation of immunotherapy. PD-L1 testing was conducted using validated anti-PD-L1 antibodies.

### Statistical analysis

Clinicopathologic and genomic data were abstracted from medical records between 2017 and 2022. A baseline NLR of ⩾5 was used to define NLR-High (NLR-H) subgroups, and NLR < 5 defined NLR-Low subgroups (NLR-L) based on previously published work showing inferior OS and PFS.^21,22^ The ORR and PFS were investigator-assessed using modified RECIST v1.1. Descriptive statistics were used to summarize categorical and continuous variables. Differences between categorical variables were evaluated using Fisher’s Exact or Chi-squared tests. Chi-squared tests included a Yates correction where sample size was < 20. Pearson–Clopper 95% confidence intervals (CIs) were reported for ORR. Differences in NLR values between patient groups were compared using Mann–Whitney *U* tests. PFS was defined as the time from the first dose of immunotherapy to the time of disease progression or death from any cause. Patients whose cancer had not progressed were censored at the time of the last imaging showing no progressive disease. OS was calculated from the time of the first dose of immunotherapy until death. Patients who were still alive at the time of data analysis were censored at the date of last follow-up/provider contact. Event-time distributions were estimated using Kaplan–Meier methods and compared with the log-rank test. Cox proportional-hazards models were used to obtain estimates of hazard ratios (HRs) in univariable and multivariable models. In the univariable analysis, variables with a signal of association with *p* < 0.1 were then included in a multivariable analysis. Statistical significance was defined as *p* < 0.05. All statistical analyses were performed using R version 4.2.2.

### Reporting guideline

The reporting of this study conforms to Strengthening the Reporting of Observational Studies in Epidemiology (STROBE: Supplemental Table 1).

## Results

### Baseline characteristics of patients with and without LM

A total of 324 patients with advanced NSCLC and no known *EGFR*, *ALK*, *ROS1*, or *RET* alterations who received PD-(L)1i as monotherapy (*N* = 323) or in combination with a CTLA-4i (*N* = 1), independent of line of therapy, were included. The median age of patients was 68 (range: 62–75), 44% were female, 81% had a history of tobacco use, and 70% had tumors with adenocarcinoma histology. PD-L1 high (PD-L1 ⩾ 50%) expression was noted in 60.4% of patients. In this study, 64 patients who were PDL1 negative received ICI monotherapy; 13 of those patients received ICI monotherapy (20.3%) as first-line therapy, and 51 (79.7%) patients in second line and beyond. In the overall cohort, brain metastases were present in 21% of patients, with no significant difference in prevalence between the two groups. Baseline characteristics of the cohort are summarized in Table 1.

**Table 1.** Clinical characteristics of patients with and without liver metastases treated with immune checkpoint inhibitors.

Characteristic	Overall *N* = 324	Liver metastasis (LM+) *N* = 59	No liver metastasis (LM−) *N* = 265	*p*-Value^a^
Age				0.090
Median (Q1–Q3)	68 (62–75)	66 (61–73)	68 (63–76)	
Treatment				0.18
PD-(L)1i monotherapy	323 (99.7%)	58 (98.3%)	265 (100.0%)	
PD-(L)1 and CTLA-4i	1 (0.3%)	1 (1.7%)	0 (0.0%)	
Sex				0.78
Female	143 (44.1%)	27 (45.8%)	116 (43.8%)	
Ethnicity				0.15
Caucasian	191 (59.0%)	29 (49.2%)	162 (61.1%)	
Asian	59 (18.2%)	16 (27.1%)	43 (16.2%)	
Other	19 (5.9%)	5 (8.5%)	14 (5.3%)	
Unknown	55 (17.0%)	9 (15.3%)	46 (17.4%)	
History of tobacco use				0.37
Yes	261 (80.6%)	50 (84.7%)	211 (79.6%)	
No	63 (19.4%)	9 (15.3%)	54 (20.4%)	
Pack years				0.73
Median (Q1–Q3)	40 (25–50)	40 (20–50)	40 (25–50)	
Unknown	66	10	56	
ECOG				0.18
PS 0–1	214 (84.3%)	39 (78.0%)	175 (85.8%)	
PS 2+	40 (15.7%)	11 (22.0%)	29 (14.2%)	
Unknown	70	9	61	
PD-L1 expression				0.30
<1%	64 (22.2%)	17 (29.8%)	47 (20.3%)	
1–49%	50 (17.4%)	9 (15.8%)	41 (17.7%)	
⩾50%	174 (60.4%)	31 (54.4%)	143 (61.9%)	
Unknown	36	2	34	
PD-L1 TPS				0.65
Median (Q1–Q3)	60 (10–90)	60 (20–90)	60 (5–90)	
Unknown	115	18	97	
Histology				0.66
Adenocarcinoma	226 (70.0%)	41 (69.5%)	185 (70.1%)	
Squamous	61 (18.9%)	13 (22.0%)	48 (18.2%)	
Other^b^	36 (11.1%)	5 (8.5%)	31 (11.7%)	
Unknown	1	0	1	
Presence of brain metastasis				0.35
Yes	65 (21%)	15 (26%)	59 (20%)	
Line of therapy				0.10
First line	189 (58.7%)	29 (49.2%)	160 (60.8%)	
⩾ Second line	133 (41.3%)	30 (50.8%)	103 (39.2%)	
Unknown	2	0	2	
Genomic alterations				0.29
KRAS non-G12C	55 (17.0%)	5 (8.5%)	50 (18.9%)	
KRAS G12C	31 (9.6%)	6 (10.2%)	25 (9.4%)	
BRAF V600E	4 (1.2%)	0 (0.0%)	4 (1.5%)	
None identified	160 (49.4%)	35 (59.3%)	125 (47.2%)	
Not assessed	48 (14.8%)	10 (16.9%)	38 (14.3%)	
Other^c^	26 (8.0%)	3 (5.1%)	23 (8.7%)	

Patients whose genomics were not assessed (48) included only those with non-adenocarcinoma histology.

a *p*-Values were estimated using Wilcoxon rank sum, Fisher’s exact, or Pearson’s Chi-squared tests.

b Other histologies included large cell neuroendocrine tumors, NSCLC not otherwise specified, and adenosquamous histology.

c Other genomic alterations included patients with MET Exon 14 skipping mutations, HER2 mutations, BRAF non-V600 mutations, and three patients with KRAS non-G12C plus BRAF non-V600 mutations.

ECOG PS, Eastern Cooperative Oncology Group Performance Status; PD-L1 TPS, programmed death ligand-1 tumor proportion score; PD-(L)1i, PD-(L)1 inhibitor; PD-(L)1i and CTLA-4i, PD-(L)1 inhibitor and CTLA-4 inhibitor; Q1, First quartile; Q3, Third quartile; TPS, Tumor Proportion Score.

We compared the clinicopathologic characteristics of patients with LM (LM+, *N* = 59) to those without (LM−, *N* = 265). Patients with LM had a similar history of tobacco use compared to patients without LM (84.7% vs 79.6%, respectively, *p* = 0.37) and had similar median pack-years of tobacco use (median 40, *p* = 0.73). Most patients had adenocarcinoma histology, 69.5% in LM+, and 70.1% in LM−. A numerically higher proportion of patients with LM received second-line ICI therapy (50.8%), compared to those without LM (39.2%, *p* = 0.1). A similar distribution of PD-L1 expression was present in both groups (*p* = 0.23), with 60.4% of patients having PD-L1 high expression. Where available, the median PD-L1 tumor proportion score (TPS) was similar in both groups (median 60, *p* = 0.65). With respect to actionable genomic alterations, *KRAS* mutations were the most common in both groups (Table 1).

Median NLR was significantly higher in patients with LM+ compared to those without (LM−) (median 7.3 (4.3–12.0) vs 4.5 (3.0–6.9), *p* < 0.001, Supplemental Figure 1). This difference remained significant in patients with adenocarcinoma histology where the median NLR was 8.3 in LM+ patients, compared with 4.0 in LM− patients (*p* < 0.001), whereas, in patients with LM and squamous histology, there was no significant difference in median NLR between LM+ and LM− patients (6.1 vs 6.3, *p* = 0.3, Supplemental Figure 2).

### Efficacy of ICI among patients with and without LM

**Figure 1.**
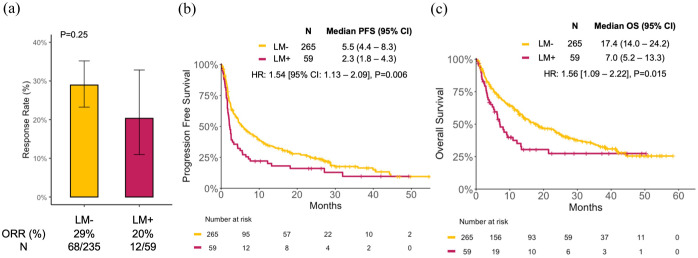
Clinical outcomes of patients with and without LM (LM+ and LM−, respectively) treated with immune checkpoint inhibitors. (a) ORR, (b) PFS, and (c) OS. CI, confidence interval; HR, hazard ratio; LM, liver metastases; ORR, objective response rate; OS, overall survival; PFS, progression-free survival.

Among the entire cohort, the ORR was 27.2% (95% CI: 22.2–32.7), the mPFS was 4.6 months (95% CI: 3.6–6.1), and the mOS was 15.1 months (12.5–19.7, Supplemental Figure 3). Compared to LM− patients, LM+ patients had lower ORR (20% vs 29%, *p* = 0.25; Figure 1(a)), significantly shorter PFS (mPFS 2.3 vs 5.5 months, HR = 1.54 (95% CI: 1.13–2.09), *p* = 0.006; Figure 1(b)), and OS (mOS 7.0 vs 17.4 months, HR = 1.56 (95% CI: 1.09–2.22), *p* = 0.015; Figure 1(c)).

**Figure 2.**
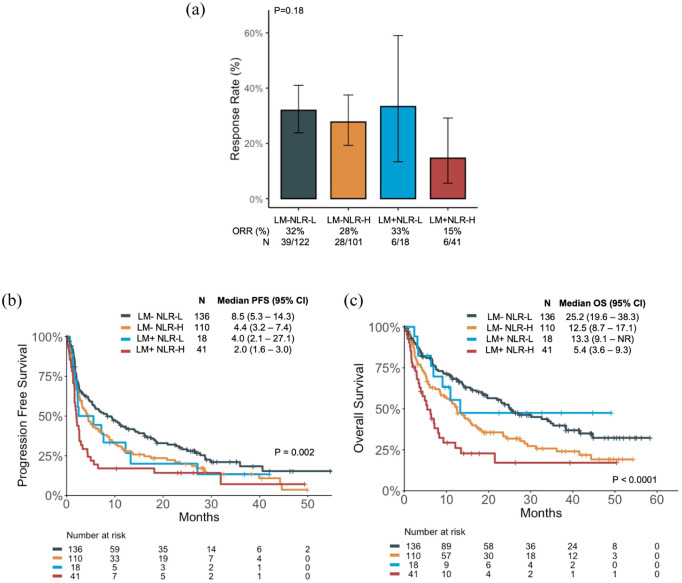
Clinical outcomes of patients with and without LM (LM+ and LM−, respectively) who are NLR High (NLR-H) and NLR Low (NLR-L). (a) ORR, (b) PFS, and (c) OS. CI, confidence interval; HR, hazard ratio; LM, liver metastases; NLR, neutrophil-to-lymphocyte ratio; ORR, objective response rate; OS, overall survival; PFS, progression-free survival.

Patients with LM who had high NLR (LM+ NLR-H) had poor ORR (15%); however, this was not significantly different from other subgroups (*p* = 0.18; Figure 2(a)). The median PFS was shortest in patients who were LM+ NLR-H (2.0 months), and longest in patients who were LM− NLR-L (8.5 months; Figure 2(b)). Similarly, patients who were LM+ NLR-H had the shortest median OS (5.4 months), and patients who were LM− NLR-L had the longest median OS (25.2 months, Figure 2(c)).

**Figure 3.**
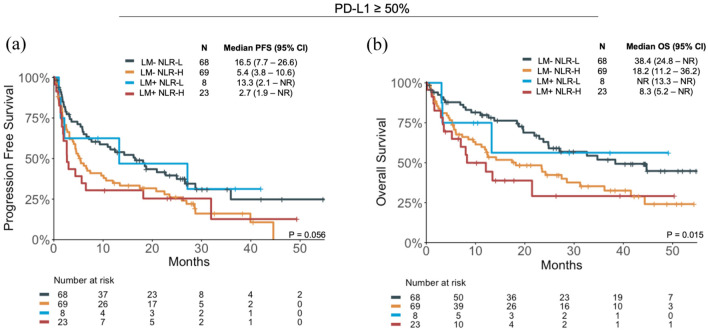
Clinical outcomes of patients in the PD-L1 high (⩾50%) subgroup, with and without LM (LM+ and LM−, respectively), who are NLR High (NLR-H) and NLR Low (NLR-L). (a) PFS and (b) OS. CI, confidence interval; HR, hazard ratio; LM, liver metastases; NLR, neutrophil-to-lymphocyte ratio; OS, overall survival; PD-L1, programmed cell death ligand 1; PFS, progression-free survival.

Patients with LM had a numerically shorter median PFS if they were NLR-H compared to NLR-L (mPFS 2.0 vs 4.0 months, HR = 1.49 (95% CI: 0.81–2.74), *p* = 0.2; Supplemental Figure 4(a)), and significantly shorter median OS (mOS 5.4 vs 13.3 months, HR = 2.46 (95% CI: 1.12–5.4), *p* = 0.02; Supplemental Figure 4(a)). Similar findings were seen in patients without LM (Supplemental Figure 4(b)). Even among the PD-L1 high subgroup of patients, those who were LM+ NLR-H continued to demonstrate the poorest outcomes; patients who were LM+ NLR-H had the shortest PFS (mPFS 2.7 months) and OS (mOS 8.3 months) among the four subgroups (Figure 3).

In univariate analysis, ECOG ⩾ 2, PD-L1 low (TPS < 50%), and receiving second-line therapy and beyond were associated with worse PFS and OS (Table 2). In addition, patients who were LM+ or LM− and NLR-High had worse PFS and OS compared to patients who were LM− and NLR-Low (Table 2). A multivariate analysis adjusting for ECOG, PD-L1 expression, and line of therapy confirmed an independent association of inferior PFS and OS in LM+ NLR-H patients compared to those who were LM− NLR-L (HR 2.39, *p* < 0.001 for PFS, and HR 3.24, *p* < 0.001 for OS, Supplemental Figure 5).

**Table 2.** Univariable analysis of clinical factors for PFS and OS.

Variable	PFS		OS	
	HR (95% CI)	*p*-Value	HR (95% CI)	*p*-Value
NLR-LM				
LM− NLR-L	Reference	—	Reference	—
LM− NLR-H	1.38 (1.04–1.84)	0.028	1.61 (1.17–2.22)	0.004
LM+ NLR-L	1.36 (0.79–2.35)	0.3	0.96 (0.46–2.00)	>0.9
LM+ NLR-H	2.07 (1.41–3.03)	<0.001	2.70 (1.75–4.15)	**<0.001**
ECOG PS				
⩾2 vs 0–1	1.74 (1.22–2.48)	0.002	1.48 (0.98–2.26)	0.065
Age				
⩾70 vs <70	1.02 (0.80–1.31)	0.9	1.15 (0.87–1.53)	0.3
Sex				
Male vs Female	1.03 (0.80–1.32)	0.8	1.16 (0.87–1.53)	0.3
Tobacco use history				
Yes vs No	0.89 (0.66–1.20)	0.4	1.31 (0.91–1.88)	0.14
Histology				
(Sq vs Non-Sq)	1.07 (0.79–1.46)	0.7	1.02 (0.72–1.46)	>0.9
PD-L1				
High (⩾50%) vs Low (<50%)	0.50 (0.38–0.65)	<0.001	0.55 (0.41–0.75)	<0.001
Line of therapy				
(⩾Second vs first)	1.44 (1.12–1.86)	0.004	1.32 (1.00–1.75)	0.052

CI, confidence interval; ECOG PS, Eastern Cooperative Oncology Group Performance Status; HR, hazard ratio; LM−, liver metastasis negative; LM+, liver metastasis positive; NLR, neutrophil-to-lymphocyte ratio; NLR-H, NLR High; NLR-L, NLR Low; PD-L1, programmed cell death ligand 1; Sq, squamous.

### Categorizing liver and extra-hepatic response

**Figure 4.**
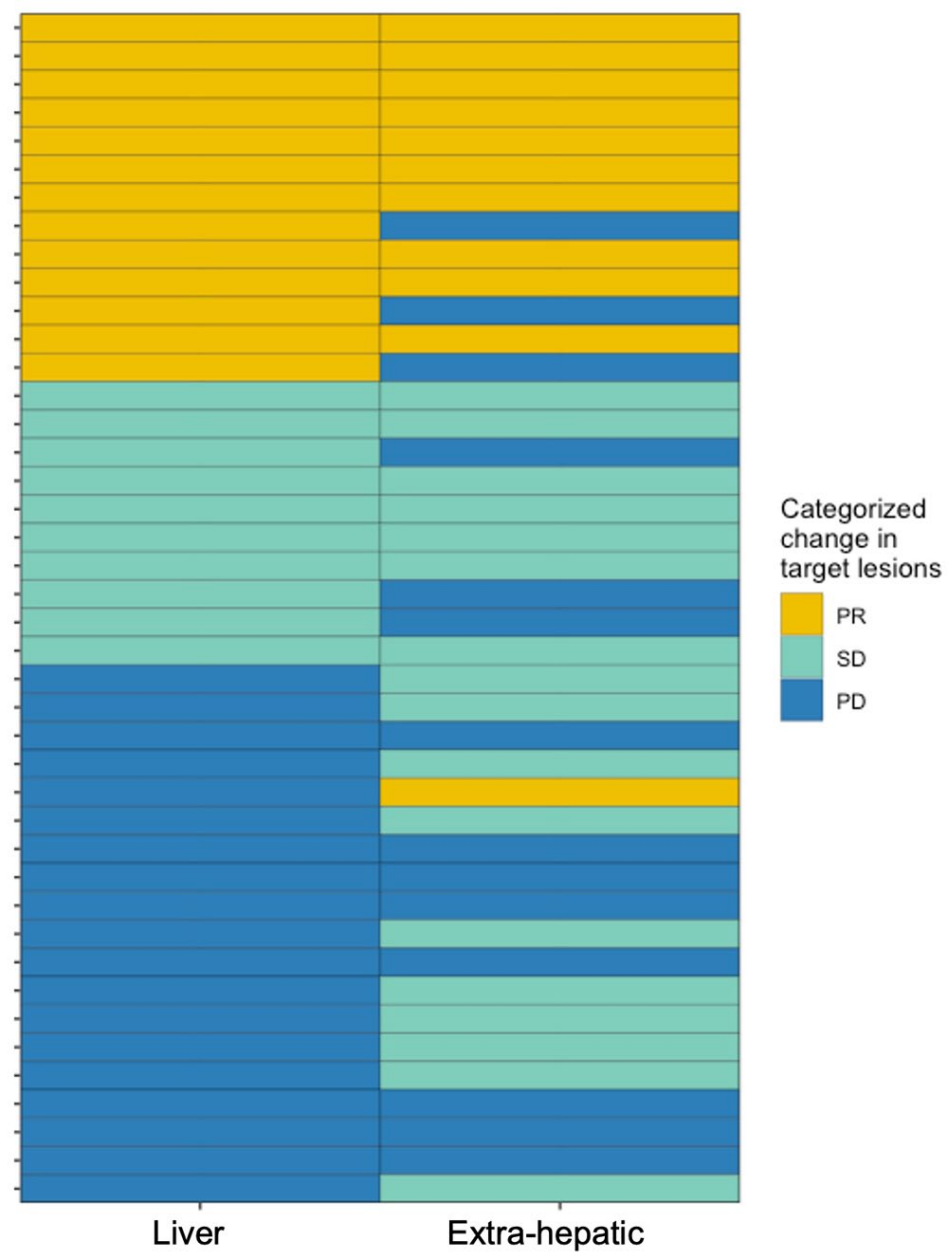
Individual patient target lesion responses in the liver and extra-hepatic sites of disease. Extra-hepatic sites included the lungs, lymph nodes, adrenal gland, spleen, and kidneys. PD, progressive disease; PR, partial response; SD, stable disease.

To investigate further whether liver response to ICI was concordant with systemic response, we conducted an analysis comparing responses in the liver to those in extra-hepatic sites, including the lung, lymph nodes, adrenal glands, and others. Discordant responses included cases where progressive disease occurred in one site while another site exhibited a partial response or stable disease. All other responses were categorized as concordant. Among 42 LM+ patients with evaluable responses, 25 patients (59.5%) had concordant responses, and 17 (40.5%) had discordant responses (Figure 4). Of the 17 discordant responders, 11 patients experienced progression in the liver, with stable disease or partial responses in an extra-hepatic site. No significant clinicopathologic differences were observed between concordant compared to discordant responders, except for a higher incidence of tobacco use in patients with concordant responses (*p* = 0.032, Table 3).

**Table 3.** Clinical characteristics of patients with concordant compared with discordant responses between hepatic and extra-hepatic sites.

Characteristic	Response		*p*-Value^a^
	Concordant, *N* = 25^a^	Discordant, *N* = 17^a^	
Age			0.92
Median (Q1–Q3)	65 (63–69)	66 (62–69)	
Sex			0.23
Female	10 (40.0%)	10 (58.8%)	
Male	15 (60.0%)	7 (41.2%)	
Ethnicity			0.81
Caucasian	14 (56.0%)	7 (41.2%)	
Asian	6 (24.0%)	6 (35.3%)	
Other	1 (4.0%)	1 (5.9%)	
Unknown	4 (16.0%)	3 (17.6%)	
History of tobacco use			0.032
Yes	24 (96.0%)	12 (70.6%)	
No	1 (4.0%)	5 (29.4%)	
Pack Years			0.63
Median (Q1–Q3)	38 (20, 50)	43 (26, 50)	
Unknown	2	5	
ECOG			0.63
PS 0–1	18 (81.8%)	11 (91.7%)	
PS ⩾2	4 (18.2%)	1 (8.3%)	
Unknown	3	5	
PD-L1 expression			>0.99
<1%	5 (21.7%)	4 (23.5%)	
1–49%	4 (17.4%)	2 (11.8%)	
⩾50%	14 (60.9%)	11 (64.7%)	
Unknown	2	0	
PD-L1 TPS			0.51
Median (Q1–Q3)	58 (15–95)	70 (50–90)	
Unknown	7	5	
Histology			0.30
Non-squamous	20 (80.0%)	11 (64.7%)	
Squamous	5 (20.0%)	6 (35.3%)	
Line			0.49
First line	13 (52.0%)	7 (41.2%)	
⩾Second line	12 (48.0%)	10 (58.8%)	
NLR			0.13
Median (Q1–Q3)	5.63 (3.50–7.89)	8.33 (6.36–11.33)	

a *p*-Values were estimated using Wilcoxon rank sum, Fisher’s exact, or Pearson’s Chi-squared tests.

ECOG PS, Eastern Cooperative Oncology Group Performance Status; NLR, neutrophil-to-lymphocyte ratio; PD-L1 TPS, programmed death ligand-1 tumor proportion score; Q1, first quartile; Q3, third quartile; TPS, tumor proportion score.

## Discussion

In this single-center study, we identified patients with advanced NSCLC who received ICIs without additional chemotherapy or anti-angiogenic therapy. We found that patients with LM had higher median NLR, and that patients with LM and who were NLR-High (NLR ⩾ 5) had the poorest outcomes independent of PD-L1 status, performance status, or line of therapy. These findings overall suggest that NLR may serve as a valuable predictor of unfavorable outcomes with ICI monotherapy, even in the presence of LM, and notably, that most responses in the liver appear concordant with extra-hepatic sites of disease.

In advanced NSCLC, several potential biomarkers of ICI response have been identified. These have included PD-L1 expression, tumor mutational burden (TMB), NLR, aneuploidy levels, and T-cell infiltration, among others. Previous work also demonstrated the utility of incorporating NLR to other clinical indicators in stratifying the prognosis of patients with NSCLC treated with immunotherapy.^22^ Higher TMB, PD-L1 score, and T-cell infiltration have correlated with improved outcomes to ICI.^23,24^ Conversely, lower NLR and lower aneuploidy levels appear to correlate with more favorable outcomes.^16,17,21,25^ In this cohort, the lack of availability of TMB did not allow additional assessment of predictors of response; however, consistent with published literature, lower NLR correlated with improved outcomes in patients with and without LM. Genomic alterations such as *KRAS* mutations, *STK11* and *KEAP1* mutations are also known to play a role in ICI efficacy.^26,27^ In our cohort, similar proportions of patients with *KRAS* alterations were present in patients with and without LM. Patients whose tumors were PDL1 negative represent a more challenging-to-treat population with poor clinical outcomes. In our study, few patients in this subgroup received ICI monotherapy; therefore, analysis in those with LM was not possible. Standard of care of therapy includes chemotherapy in combination with ICI as per KEYNOTE-189,^28^ CheckMate 9LA,^29^ or POSEIDON^30^ trials; however, novel PD1-VEGF bispecifics such as ivoniscemab have shown some efficacy in early-phase trials in those with PDL1-negative tumors.^31^

Patients with LM are known to have worse prognosis and outcomes to systemic therapy, in particular to ICI compared to patients without LM.^32^ Here, we identified that among patients with LM, NLR predicted for worse outcomes to ICI monotherapy. One hypothesis that may account for worse outcomes of ICI is that the tolerogenic immune microenvironment of the liver may both resist anti-tumor immunity locally as well as be preferentially colonized by less immunogenic tumors. The presence of tolerogenic resident immune cell populations may play a role in modulating the T-cell response in patients receiving immunotherapy and potentially contribute to inducing systemic tolerance through cytotoxic T-cell deletion.^33,34^ There may also be a unique synergy between LM and activation of the tumor-myeloid axis, which leads to particularly immunotherapy-refractory disease.^14,15,34^ In particular, LM may stimulate local production of inflammatory cytokines, including IL-6, G-CSF, CCL2, and TGF-B, driving systemic myelopoiesis, neutrophil expansion, and suppression of lymphocyte activity.^35,36^ In addition, LMs enrich for granulocytic myeloid-derived suppressor cells (gMDSCs), which further impair T-cell function and promote immune evasion.^34^ These effects contribute to both peripheral lymphopenia and neutrophilia, leading to NLR expansion and resistance to ICIs. It is also possible that metastatic sites of disease carry unique mutational signatures not present in the primary tumor that may account for poor responses; however, the high proportion of concordant responders in this cohort may argue against this hypothesis.

Limitations of this study include its retrospective nature and single-center data availability, with a limited number of patients with LM receiving ICIs. Identifying reliable, consistent biomarkers of response to ICI presents an ongoing challenge to the field. While NLR is one of many potential biomarkers, our study delved into a particular subset of patients with less favorable outcomes, examining the predictive potential of an easily measurable biomarker. Further stratification of this biomarker rather than the use of a cutoff may improve upon its performance. There is also evidence that dynamic changes in NLR may be better predictors of response.^37^ Additional information regarding the extent of disease could shed more light on the outcomes for patients with LM, especially since a higher NLR has been observed in patients with a greater burden of disease.^38^

Patients with LM who were NLR-High and who had high PD-L1 expression in this cohort still had inferior clinical outcomes compared to patients without LM. This may argue for the use of combination chemo-immunotherapy in these patients despite high PD-L1 expression, with some data suggesting similar efficacy of combination therapy in patients with compared to without LM.^28^ Emerging data also suggest that the addition of anti-angiogenic therapy may result in improved ICI efficacy. Preclinical data suggest that vascular endothelial growth factor (VEGF) inhibition promotes cytotoxic T-cell infiltration to help overcome the immune tolerant environment of the liver.^39^ The efficacy of combining a VEGF inhibitor with immune checkpoint blockade was reported in a subgroup analysis of IMpower150, which showed improved outcomes in patients with LM receiving combination therapy (atezolizumab/bevacizumab/carboplatin/paclitaxel vs bevacizumab/carboplatin/paclitaxel, HR 0.68, median OS 13.2 months vs 9.1 months).^40^

Lastly, we explored the hepatic and extra-hepatic response of patients with LM and found that most responses are concordant. In patients with melanoma, there are data showing that CD8+ T cells are similarly depleted in extra-hepatic and hepatic metastases, suggestive of a more systemic effect at play.^8^ We also identified a subgroup of patients who had an extra-hepatic response and progressive disease in the liver. This raises the possibility that the liver has a more unique tumor immune microenvironment. Additional translational work through immune cell profiling would be valuable to explore how the systemic versus liver-specific tumor immune microenvironment might vary.

## Conclusion

In conclusion, our study demonstrates that patients with advanced NSCLC and LM who have high NLR derive less benefit from ICI, and biomarkers that associate with ICI response in this subgroup remain limited. Future translational work focused on understanding the tumor-myeloid axis in patients with LM may help identify novel biomarkers and treatment strategies.

## Supplemental Material

sj-docx-6-tam-10.1177_17588359251367315 – Supplemental material for A retrospective analysis exploring the association of pretreatment neutrophil-to-lymphocyte ratio and immune checkpoint inhibitor outcomes in patients with advanced NSCLC and liver metastases Supplemental material, sj-docx-6-tam-10.1177_17588359251367315 for A retrospective analysis exploring the association of pretreatment neutrophil-to-lymphocyte ratio and immune checkpoint inhibitor outcomes in patients with advanced NSCLC and liver metastases by Maisam Makarem, Tuan Hoang, Mitchell J. Elliott, Andrew Rabinovitch, Katrina Hueniken, Shelley Kuang, Sabine Schmid, Ming Sound Tsao, Tracy McGaha, Pamela S. Ohashi, Frances A. Shepherd, Penelope A. Bradbury, Geoffrey Liu, Natasha B. Leighl, Adrian Sacher and Sally C. M. Lau in Therapeutic Advances in Medical Oncology

sj-jpg-1-tam-10.1177_17588359251367315 – Supplemental material for A retrospective analysis exploring the association of pretreatment neutrophil-to-lymphocyte ratio and immune checkpoint inhibitor outcomes in patients with advanced NSCLC and liver metastases Supplemental material, sj-jpg-1-tam-10.1177_17588359251367315 for A retrospective analysis exploring the association of pretreatment neutrophil-to-lymphocyte ratio and immune checkpoint inhibitor outcomes in patients with advanced NSCLC and liver metastases by Maisam Makarem, Tuan Hoang, Mitchell J. Elliott, Andrew Rabinovitch, Katrina Hueniken, Shelley Kuang, Sabine Schmid, Ming Sound Tsao, Tracy McGaha, Pamela S. Ohashi, Frances A. Shepherd, Penelope A. Bradbury, Geoffrey Liu, Natasha B. Leighl, Adrian Sacher and Sally C. M. Lau in Therapeutic Advances in Medical Oncology

sj-jpg-2-tam-10.1177_17588359251367315 – Supplemental material for A retrospective analysis exploring the association of pretreatment neutrophil-to-lymphocyte ratio and immune checkpoint inhibitor outcomes in patients with advanced NSCLC and liver metastases Supplemental material, sj-jpg-2-tam-10.1177_17588359251367315 for A retrospective analysis exploring the association of pretreatment neutrophil-to-lymphocyte ratio and immune checkpoint inhibitor outcomes in patients with advanced NSCLC and liver metastases by Maisam Makarem, Tuan Hoang, Mitchell J. Elliott, Andrew Rabinovitch, Katrina Hueniken, Shelley Kuang, Sabine Schmid, Ming Sound Tsao, Tracy McGaha, Pamela S. Ohashi, Frances A. Shepherd, Penelope A. Bradbury, Geoffrey Liu, Natasha B. Leighl, Adrian Sacher and Sally C. M. Lau in Therapeutic Advances in Medical Oncology

sj-jpg-3-tam-10.1177_17588359251367315 – Supplemental material for A retrospective analysis exploring the association of pretreatment neutrophil-to-lymphocyte ratio and immune checkpoint inhibitor outcomes in patients with advanced NSCLC and liver metastases Supplemental material, sj-jpg-3-tam-10.1177_17588359251367315 for A retrospective analysis exploring the association of pretreatment neutrophil-to-lymphocyte ratio and immune checkpoint inhibitor outcomes in patients with advanced NSCLC and liver metastases by Maisam Makarem, Tuan Hoang, Mitchell J. Elliott, Andrew Rabinovitch, Katrina Hueniken, Shelley Kuang, Sabine Schmid, Ming Sound Tsao, Tracy McGaha, Pamela S. Ohashi, Frances A. Shepherd, Penelope A. Bradbury, Geoffrey Liu, Natasha B. Leighl, Adrian Sacher and Sally C. M. Lau in Therapeutic Advances in Medical Oncology

sj-jpg-4-tam-10.1177_17588359251367315 – Supplemental material for A retrospective analysis exploring the association of pretreatment neutrophil-to-lymphocyte ratio and immune checkpoint inhibitor outcomes in patients with advanced NSCLC and liver metastases Supplemental material, sj-jpg-4-tam-10.1177_17588359251367315 for A retrospective analysis exploring the association of pretreatment neutrophil-to-lymphocyte ratio and immune checkpoint inhibitor outcomes in patients with advanced NSCLC and liver metastases by Maisam Makarem, Tuan Hoang, Mitchell J. Elliott, Andrew Rabinovitch, Katrina Hueniken, Shelley Kuang, Sabine Schmid, Ming Sound Tsao, Tracy McGaha, Pamela S. Ohashi, Frances A. Shepherd, Penelope A. Bradbury, Geoffrey Liu, Natasha B. Leighl, Adrian Sacher and Sally C. M. Lau in Therapeutic Advances in Medical Oncology

sj-jpg-5-tam-10.1177_17588359251367315 – Supplemental material for A retrospective analysis exploring the association of pretreatment neutrophil-to-lymphocyte ratio and immune checkpoint inhibitor outcomes in patients with advanced NSCLC and liver metastases Supplemental material, sj-jpg-5-tam-10.1177_17588359251367315 for A retrospective analysis exploring the association of pretreatment neutrophil-to-lymphocyte ratio and immune checkpoint inhibitor outcomes in patients with advanced NSCLC and liver metastases by Maisam Makarem, Tuan Hoang, Mitchell J. Elliott, Andrew Rabinovitch, Katrina Hueniken, Shelley Kuang, Sabine Schmid, Ming Sound Tsao, Tracy McGaha, Pamela S. Ohashi, Frances A. Shepherd, Penelope A. Bradbury, Geoffrey Liu, Natasha B. Leighl, Adrian Sacher and Sally C. M. Lau in Therapeutic Advances in Medical Oncology

## Declarations

**Ethics approval and consent to participate:** Ethics approval and consent to participate in the study were approved by the Institutional Review Board of the University Health Network. Approval numbers: IRB 20-5935 and 18-5450. Formal consent was waived due to the retrospective nature of the study. All individual-level data were anonymized and were only available to authorized researchers.

**Consent for publication:** Not applicable.

**Competing interests:** Maisam Makarem, MD, PhD has received honoraria or consultant fees from Pfizer, Merck, and other support (travel/accommodation/meals) from AstraZeneca, Boehringer Ingelheim, Pfizer, and Merck. Sabine Schmid, MD, has received funding (institution) from AstraZeneca, Sanofi, Bristol Meyer Swibb, Merck, Janssen, Roche, Pfizer, Regeneron, and MSD, and funding (personal) from Roche and Amgen. Pamela S. Ohashi, PhD, is on the Scientific Advisory Board for Providence Therapeutics, Tikva Allocell, and Rondo Therapeutics, Inc., and holds a sponsored research agreement with Providence Therapeutics. Geoffrey Liu, MD, has received honoraria, grant funding (to his institution), and/or participated in advisory boards of Adela, Amgen, AstraZeneca, Bayer, Boehringer Ingelheim, EMD Serono, Gilead, GlaxoKlineSmith, Jazz Pharmaceuticals, Lilly, Merck, Novartis, Nuvation Bio, OxCan, Pfizer, and Takeda, and grant funding from US NCI, US NIDCR, CIHR, CCSRI, TFRI, Lung Cancer Canada, and Princess Margaret Cancer Foundation. Natasha B. Leighl, MD, MMSc is on the Data Safety Monitoring Board (uncompensated) for Mirati Therapeutics and Daiichi Sankyo, has received research funding (institution) from Amgen, AstraZeneca, BMS, Eli Lilly, GlaxoSmithKline, Janssen, MSD, Novartis, Pfizer, Roche, Takeda, Guardant Health, Inivata/Neogenomics, and travel accommodation (CME lectures) from AstraZeneca, MSD, Roche, Janssen, Sanofi, Guardant Health, Pfizer, DavaOncology. Adrian Sacher, MD, MMSc, is a member of the advisory committee (no personal fees) for Genentech, Amgen, and Merck, has received institutional research funding from AstraZeneca, Amgen, Genetech, Merck, Lilly, Bristol Meyers Swib, Sepctrum, GSK, Iovance, CRISPR Therapeutics, BridgeBio, HotSpot Therapeutics, AdaptImmune, and travel expenses for clinical trial investigator meetings from Amgen, Merck, and Genentech-Roche. Sally CM Lau, MD, MPH, is an employee of AstraZeneca and a member of the advisory board of AstraZeneca. The remaining authors have no conflict of interest to declare.

**Availability of data and materials:** The datasets used and/or analyses during the current study are available from the corresponding authors upon request.
