# Supplementary figures and images for: A retrospective analysis exploring the association of pretreatment neutrophil-to-lymphocyte ratio and immune checkpoint inhibitor outcomes in patients with advanced NSCLC and liver metastases
Source: Ther Adv Med Oncol. 2025 Oct 22;17:17588359251367315. doi: 10.1177/17588359251367315 (PMC12559634; doi:10.1177/17588359251367315)

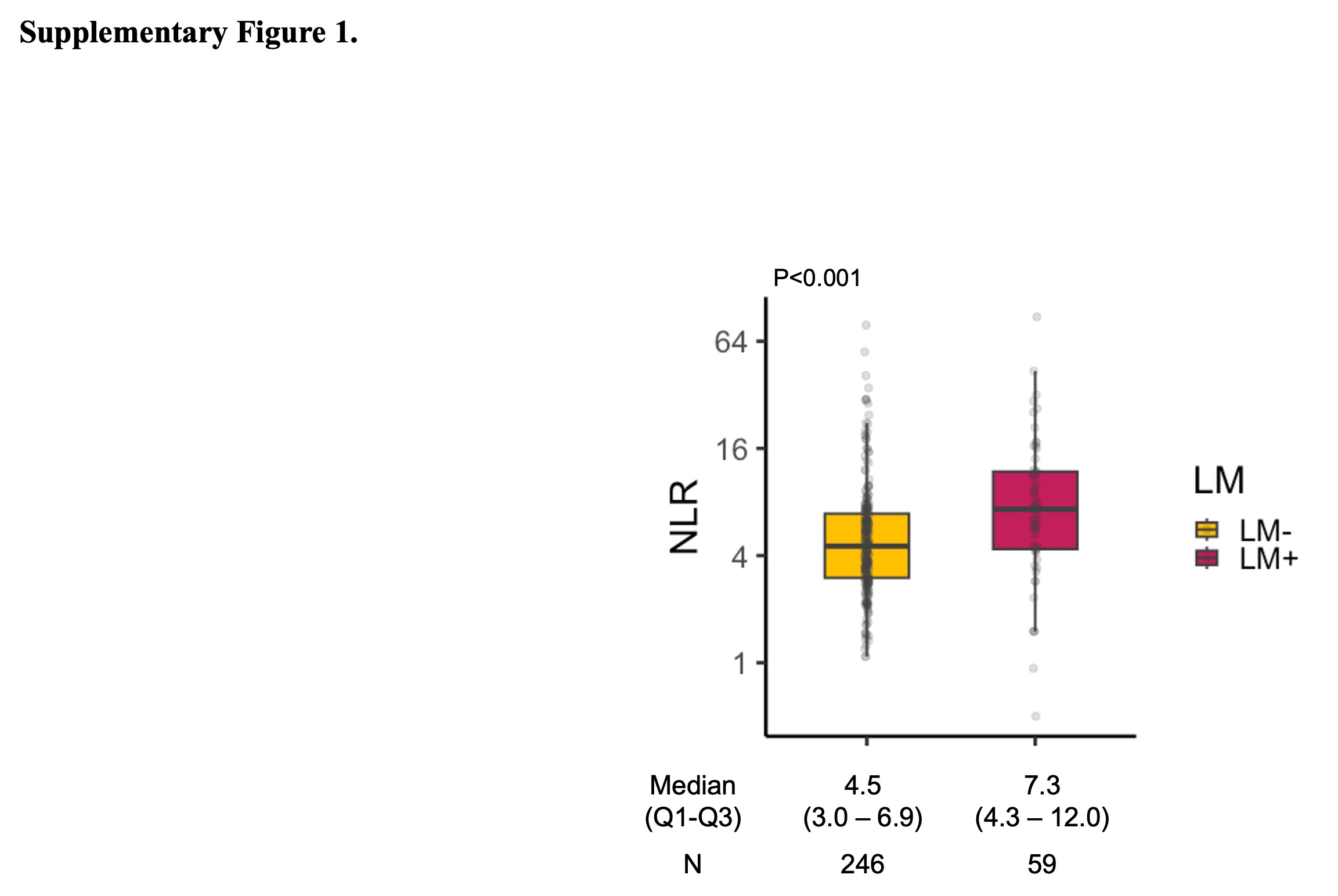

Supplement: sj-jpg-1-tam-10.1177_17588359251367315 – Supplemental material for A retrospective analysis exploring the association of pretreatment neutrophil-to-lymphocyte ratio and immune checkpoint inhibitor outcomes in patients with advanced NSCLC and liver metastases [file sj-jpg-1-tam-10.1177_17588359251367315.jpg]

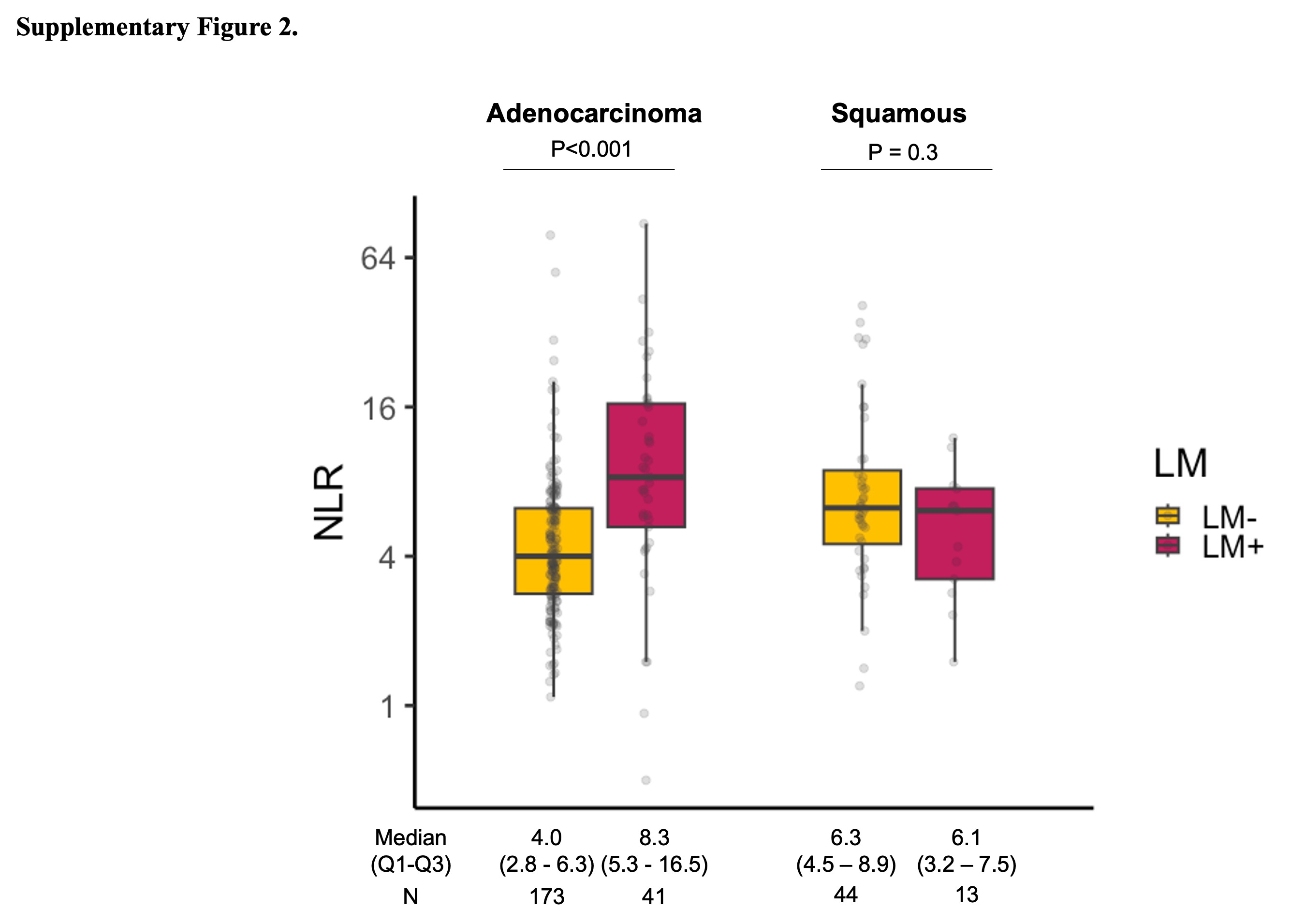

Supplement: sj-jpg-2-tam-10.1177_17588359251367315 – Supplemental material for A retrospective analysis exploring the association of pretreatment neutrophil-to-lymphocyte ratio and immune checkpoint inhibitor outcomes in patients with advanced NSCLC and liver metastases [file sj-jpg-2-tam-10.1177_17588359251367315.jpg]

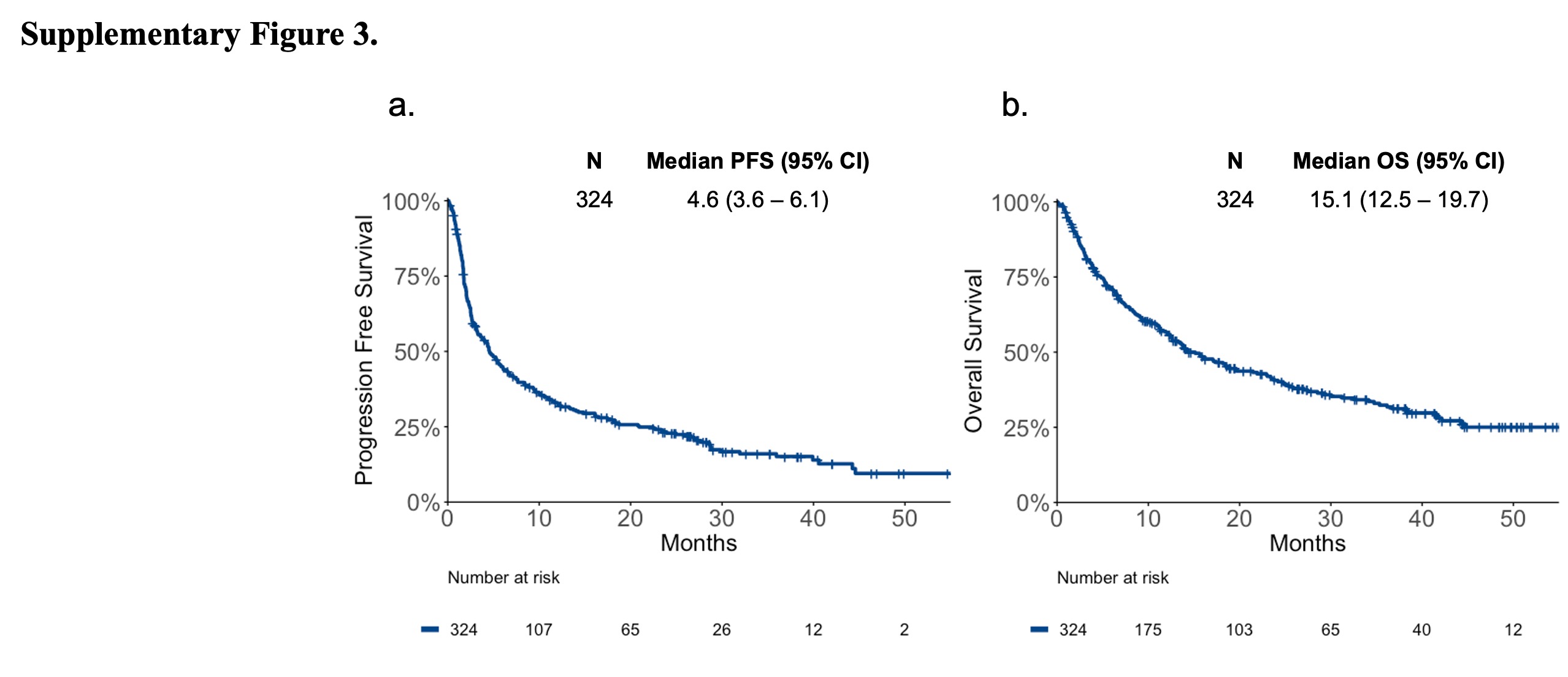

Supplement: sj-jpg-3-tam-10.1177_17588359251367315 – Supplemental material for A retrospective analysis exploring the association of pretreatment neutrophil-to-lymphocyte ratio and immune checkpoint inhibitor outcomes in patients with advanced NSCLC and liver metastases [file sj-jpg-3-tam-10.1177_17588359251367315.jpg]

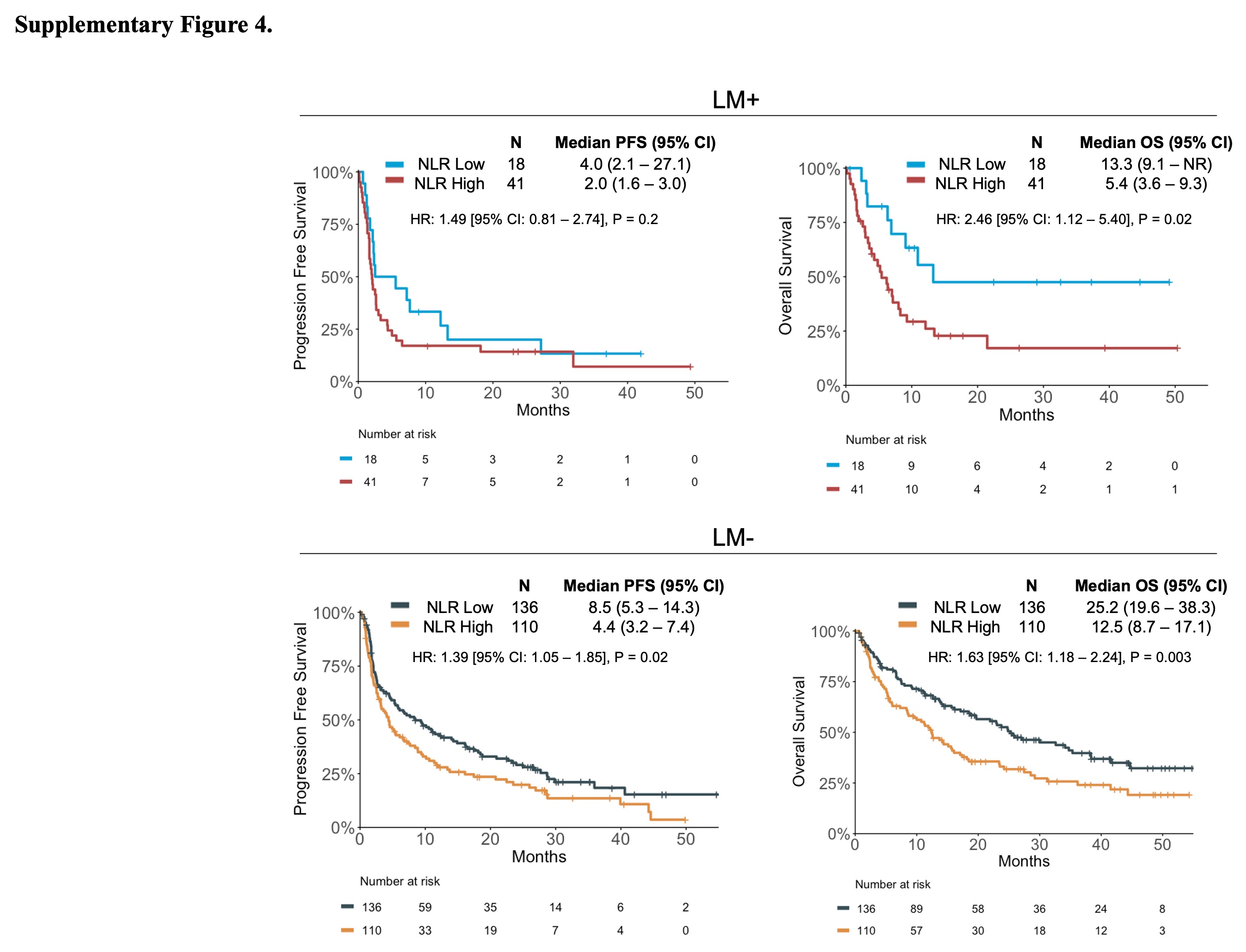

Supplement: sj-jpg-4-tam-10.1177_17588359251367315 – Supplemental material for A retrospective analysis exploring the association of pretreatment neutrophil-to-lymphocyte ratio and immune checkpoint inhibitor outcomes in patients with advanced NSCLC and liver metastases [file sj-jpg-4-tam-10.1177_17588359251367315.jpg]

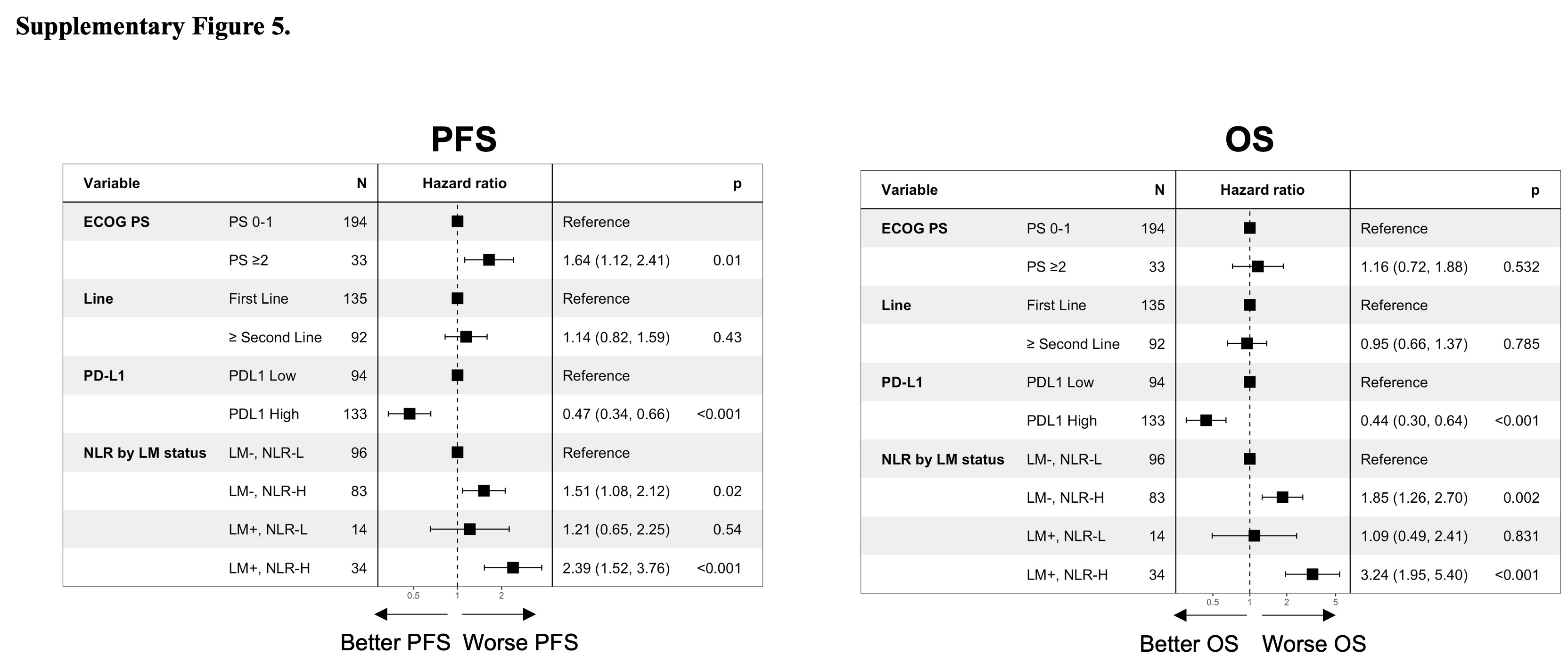

Supplement: sj-jpg-5-tam-10.1177_17588359251367315 – Supplemental material for A retrospective analysis exploring the association of pretreatment neutrophil-to-lymphocyte ratio and immune checkpoint inhibitor outcomes in patients with advanced NSCLC and liver metastases [file sj-jpg-5-tam-10.1177_17588359251367315.jpg]
